# Health Care Cost Concerns and Hardships for Families of Children With Disabilities

**DOI:** 10.1001/jamanetworkopen.2025.7826

**Published:** 2025-04-24

**Authors:** Amy J. Houtrow, Chloe S. Shearer, Gina McKernan, Karen Kuhlthau, Rishi Agrawal

**Affiliations:** 1 Department of Physical Medicine and Rehabilitation, School of Medicine, University of Pittsburgh, Pittsburgh, Pennsylvania; 2 Department of Pediatrics, University of Pittsburgh School of Medicine, Pittsburgh, Pennsylvania; 3 Department of Pediatrics, Harvard Medical School, Boston, Massachusetts; 4 Division of General Academic Pediatrics, Department of Pediatrics, Massachusetts General Hospital for Children, Boston; 5 Department of Pediatrics, Feinberg School of Medicine, Northwestern University, Chicago, Illinois

## Abstract

This survey study uses data from the 2019-2022 National Health Interview Survey to estimate heath care–related financial hardships experienced by families of children with disabilities.

## Introduction

Families of children with disabilities report more health care–related financial problems than families of children without disabilities.^1^ Their families are also more likely to report inadequate insurance coverage.^2,3^ We used the National Health Interview Survey (NHIS) to quantify the hardships of health care costs to families of children with disabilities in terms of difficulty paying their child’s medical bills, concerns about paying their child’s health care bills, and their child’s care delayed or forgone due to cost.

## Methods

Data from the 2019-2022 NHIS for children aged 5 to 17 years were pooled for these analyses (eAppendix in Supplement 1). The NHIS is a nationally representative cross-sectional survey of the noninstitutionalized US population conducted by the National Center for Health Statistics.^4^ Information about the household’s sample child was collected via computer-assisted personal interview with a parent or guardian knowledgeable about the child’s health and disabilities, if present.^5^ Statistical analysis was performed from December 2024 to February 2025. Prevalence estimates and 95% CIs were calculated with survey weights applied. The χ^2^ test was applied to determine within-group prevalence differences, and the Cramer *V* was calculated for effect size estimates. The main outcome was the presence of health care–related financial hardship measured by caregiver report of difficulty paying the child’s medical bills, concerns about paying the child’s medical bills, delayed or forgone medical care due to cost and/or delayed or forgone prescriptions due to cost. Outcome estimates were adjusted for sociodemographic factors with missing data deleted. All *P* values were from 2-sided tests, and results were deemed statistically significant at *P* < .05. All analyses were conducted using R software, version 4.4.1. This secondary analysis falls under the exempt category for the University of Pittsburgh institutional review board because the data are publicly available. The study follows the AAPOR reporting guideline for survey studies.

## Results

Among the 22 670 children aged 5 to 17 years in the analytic sample representing 53 586 children annually, the overall prevalence of disability among children aged 5 to 17 years was 17.4% (95% CI, 16.7%-18.1%) (Table 1). The most commonly reported disabilities were emotional or behavioral (10.8% [95% CI, 10.2%-11.3%]). Disability prevalence varied across sociodemographic characteristics, with generally weak associations (Table 1). Children with disabilities were more likely to have public insurance (adjusted odds ratio [AOR], 1.42 [95% CI, 1.26-1.60]) or a combination of private and public insurance (AOR, 2.79 [95% CI, 2.10-3.71]) than their peers without disabilities (Table 2). Families of children with disabilities were almost twice as likely to experience any of 6 financial hardships than families of children without disabilities (22.3% [95% CI, 22.3%-20.7%] vs 12.6% [95% CI, 12.0%-13.3%]; AOR, 1.91 [95% CI, 1.70-2.14]). Families of children with disabilities had more difficulty paying medical bills (AOR, 1.97, 95% CI 1.76-2.21) and were more likely to be very worried about medical expenses (AOR, 1.35 [95% CI, 1.18-1.55]). These families also experienced higher rates of delayed and forgone care due to cost compared with families of children without disabilities.

**Table 1.** Prevalence of Disability in Children Aged 5 to 17 Years by Sociodemographic Characteristics (N = 22 670)

Characteristic	Weighted % of population (95% CI)	χ^2^ test	*P* value	Cramer *V*
Presence of disability among all children^a^	17.4 (16.7-18.1)	NA	NA	NA
Disability subtypes (a child could have >1 type)				
Sensory	0.6 (0.5-0.8)	NA	NA	NA
Mobility	0.8 (0.7-1.0)			
Communication or cognition	3.2 (2.9-3.5)			
Self-care	0.6 (0.5-0.7)			
Emotional or behavioral	10.8 (10.2-11.3)			
Learning or developmental	9.8 (9.3-10.3)			
Age, y				
5-11	16.6 (15.6-17.5)	7.1	.008	0.02
12-17	18.2 (17.3-19.1)			
Sex	19.3 (18.3-20.3)			
Male	15.3 (14.4-16.3)	36.1	<.001	0.05
Female	19.3 (18.3-20.3)			
Race and ethnicity				
Hispanic	15.9 (14.6-17.2)	18.4	<.001	0.06
Non-Hispanic American Indian or Alaska Native	22.7 (16.8-28.6)			
Non-Hispanic Asian	8.2 (6.4-9.9)			
Non-Hispanic Black or African American	19.2 (16.9-21.5)			
Non-Hispanic White	18.2 (17.3-19.2)			
Other^b^	17.8 (14.6-21.0)			
Nativity				
US	17.7 (16.9-18.4)	12.9	<.001	0.03
Outside US	12.6 (9.9-15.3)			
Family income as % of federal poverty level				
<50	27.5 (26.0-28.6)	4.6	<.001	0.11
50-99	24.9 (24.1-25.6)			
100-199	19.0 (18.4-19.6)			
200-299	15.5 (14.8-16.1)			
300-399	14.8 (14.0-15.4)			
400-499	16.0 (14.8-17.1)			
≥500	13.4 (13.0-13.8)			
Rurality				
Nonrural	17.0 (16.2-17.7)	6.4	.01	0.02
Rural	19.8 (17.8-21.7)			

^a^ Disability identified if the respondent reported the child had “a lot of difficulty” or “cannot do it at all” for 1 or more of the core functioning domains of seeing, hearing, mobility, self-care, communication, learning, remembering, concentrating, accepting change, controlling behavior or making friends, and/or had daily anxiety or depression or currently had autism spectrum disorder, intellectual disability, or a learning disability.

^b^ Included other single and multiple races (specific races not available in the public files).

**Table 2.** Health Care Costs, Concerns, and Hardships for Children With Disabilities Compared With Children Without Disabilities

Characteristic	Finding among children, % (95% CI)		Adjusted odds ratio (95% CI)^a^
	With disabilities	Without disabilities	
Type of insurance			
Private only	42.7 (40.4-45.0)	56.5 (55.1-57.9)	0.64 (0.57-0.72)
Public only	49.1 (46.9-51.2)	36.4 (35.1-37.7)	1.42 (1.26-1.60)
Private and public	4.4 (3.4-5.4)	1.5 (1.3-1.7)	2.79 (2.10-3.71)
Uninsured	3.8 (3.1-4.5)	5.6 (5.1-6.2)	0.67 (0.54-0.82)
Financial hardships			
Family reported difficulty paying medical bills	19.9 (18.3-21.5)	11.2 (10.6-11.8)	1.97 (1.76-2.21)
Family reported being very worried about paying medical bills if child got sick or had an accident	11.7 (10.3-13.0)	8.9 (8.2-9.6)	1.35 (1.18-1.55)
Delayed child’s medical care due to cost in past year	2.4 (1.8-3.1)	1.1 (0.9-1.3)	2.20 (1.68-3.16)
Child’s medical care forgone due to cost in past year	2.3 (1.7-2.9)	0.9 (0.7-1.1)	2.64 (1.89-3.69)
Delayed child’s prescriptions due to cost in past year^b^	1.4 (0.9-1.8)	0.6 (0.4-0.7)	1.55 (1.02-2.34)
Child’s prescriptions forgone due to cost in past year	2.4 (1.7-3.0)	0.9 (0.7-1.1)	2.73 (1.96-3.80)
Any of the 6 hardships	22.3 (20.7-23.9)	12.6 (12.0-13.3)	1.91 (1.70-2.14)

^a^ Adjusted for sociodemographic characteristics that were statistically associated with the outcomes and not collinear (race and ethnicity, born in US, federal poverty level, and rurality). All adjusted odds ratios are statistically significant (*P* < .05).

^b^ The χ^2^ for this comparison was not significant (*P* = .16), but the adjusted odds ratio was. All other χ^2^ comparisons were statistically significant (*P* < .05).

## Discussion

This study contributes to the existing literature by identifying that while insurance coverage is higher among children with disabilities, their families had higher adjusted odds for all of the financial hardships evaluated, compared with families of children without disabilities. This finding suggests that insurance is inadequate for disabled children. A key component of adequate insurance is the lack out-of-pocket expenses or having out-of-pocket expenses that were usually or always reasonable, such as for copays and coinsurance for diagnostic tests, visits, services, and treatments.^3^ Proposed cuts to the federal contribution to Medicaid^6^ would likely exacerbate the financial distress of these families. These data demonstrate a need to structure health insurance policies to ensure that children with disabilities have their needed medical care covered in a way that is not financially burdensome to families.^3^ This study is limited by the cross-sectional nature of the survey design, which does not allow for assessment of causality. Nonetheless, recognizing that nearly one-fourth of families of children with disabilities face financial hardships, worry about the cost of health care, and/or delay or forgo health care for their children with disabilities should be a call to action to improve insurance adequacy.

## Notes

Supplement 1.
eAppendix.

eReferences.

Supplement 2.
Data Sharing Statement
