# Supplementary material for: Health Care Cost Concerns and Hardships for Families of Children With Disabilities
Source: JAMA Netw Open. 2025 Apr 24;8(4):e257826. doi: 10.1001/jamanetworkopen.2025.7826 (PMC12022804; doi:10.1001/jamanetworkopen.2025.7826)
Supplement: Supplement 1. — eAppendix. eReferences. [file jamanetwopen-e257826-s001.pdf]

Supplementary Online Content

Houtrow AJ, Shearer CS, McKernan G, Kuhlthau K, Agrawal R. Health care cost concerns and hardships for families of children with disabilities. *JAMA Netw Open.* 2025;8(4):e257826. doi:10.1001/jamanetworkopen.2025.7826

eAppendix.

eReferences.

This supplementary material has been provided by the authors to give readers additional information about their work.

© 2025 Houtrow AJ et al. *JAMA Network Open.*

eAppendix.

The response rates for each year (highest in 2019 at 59.1% and lowest in 2022 at 45.8%) were calculated by dividing the number of interviewed caregivers for sample children by the number of eligible sample children from households with completed rosters, and then multiplying this by the household roster completion rate.[1-4] A sample weight variable is included in each year's datafile calculated to account for the probability of non-selection of minority groups, as well as nonresponse bias among selected households.[5]

© 2025 Houtrow AJ et al. *JAMA Network Open.*

eReferences.

1. National Center for Health Statistics. 2019 Survey Description National Health Interview Survey *https://ftpcdcgov/pub/health_Statistics/NCHs/Dataset_Documentation/NHIS/2019/srvydesc-508pdf*. 2020 Accessed 2/7/25
2. National Center for Health Statistics. 2020 Survey Description National Health Interview Survey *https://ftpcdcgov/pub/health_statistics/nchs/dataset_documentation/NHIS/2020/srvydesc-508pdf*. 2021;accessed 2/7/25
3. National Center for Health Statistics. 2021 Survey Description National Health Interview Survey *https://ftpcdcgov/pub/Health_Statistics/NCHS/Dataset_Documentation/NHIS/2021/srvydesc-508pdf*. 2022;accessed 2/7/25
4. National Center for Health Statistics. 2022 Survey Description National Health Interview Survey *https://ftpcdcgov/pub/health_Statistics/nchs/Dataset_Documentation/NHIS/2022/srvydesc-508pdf*. 2023;accessed 2/7/25
5. Moriarity C, Parsons VL, Jonas K, Schar BG, Bose J, Bramlett MD. Sample design and estimation structures for the National Health Interview Survey, 2016–2025. *Centers for Disease Control and Prevention*. 2022;accessed 2.25.25(https://stacks.cdc.gov/view/cdc/115394)

© 2025 Houtrow AJ et al. *JAMA Network Open.*
