# Supplementary material for: Health Care Cost Concerns and Hardships for Families of Children With Disabilities
Source: JAMA Netw Open. 2025 Apr 24;8(4):e257826. doi: 10.1001/jamanetworkopen.2025.7826 (PMC12022804; doi:10.1001/jamanetworkopen.2025.7826)
Supplement: Supplement 2. — Data Sharing Statement [file jamanetwopen-e257826-s002.pdf]

Data Sharing Statement

Houtrow. Health Care Cost Concerns and Hardships for Families of Children With Disabilities. *JAMA Netw Open*. Published April 24, 2025. doi:10.1001/jamanetworkopen.2025.7826

Data
Data available: No

Additional Information
Explanation for why data not available: it is from a publicly available dataset
